# Supplementary material for: Predicting Diagnosis of Australian Canine and Feline Urinary Bladder Disease Based on Histologic Features
Source: Vet Sci. 2020 Nov 27;7(4):190. doi: 10.3390/vetsci7040190 (PMC7712252; doi:10.3390/vetsci7040190)
Supplement: Supplementary file 1 [file vetsci-07-00190-s001.pdf]

Supplementary material

Supplementary Table 1: All animal, sampling and diagnostic histological variables measured on each bladder slide and used in the logistic regression modeling process.

Variable label	Variable description	Level	Value label
**Animal or source variables**			
Species	Species	1	Feline
		2	Canine
Source	Source of sample	1	UQVLS
		2	MUSVM
		3	Prospective sampling
**Sampling variables**			
Full_thickness	Full thickness sample (outer detrusor muscle included)	1	No
		2	Yes
Uroth_denud	Percent of urothelium that has been denuded	1	All intact (no denudation)
		2	1-25% of urothelium has been denuded
		3	26-50%
		4	51-99%
		5	100%
Uroth_artefact	Urothelial artefact causing denudation	1	No
		2	Yes
Edema_artefact	SM edema artefact	1	No
		2	Yes
**Diagnosis variables**			
Uroth_ulcer	Urothelial ulceration	1	No
		2	Yes
Uroth_hyperp	Urothelial hyperplasia	1	No
		2	Yes
Uroth_react	Reactive urothelium	1	No

		2	Yes
Neoplastic	Neoplastic urothelium	1	No
		2	Yes
Neo_inv	SM neoplastic invasion	1	No
		2	Yes
Uroth_inflamm	Amount of urothelium that was being infiltrated by inflammatory cells	1	None
		2	1-25%
		3	26-50%
		4	>50%
SM_hem	SM hemorrhage amount	1	No SM hemorrhage (none)
		2	Hemorrhage present in up to 25% of the SM (mild)
		3	Hemorrhage present in 26-50% of the SM (moderate)
		4	Hemorrhage present in >50% of the SM (severe)
SM_cong	SM congestion	1	No
		2	Yes
SM_edema	SM edema	1	No
		2	Yes
SM_inflamm	SM inflammation amount	1	None
		2	Occasional inflammatory cell per LPF (100x magnification)
		3	<100 inflammatory cells per LPF
		4	>100 inflammatory cells per LPF
SM_inflamm_type	SM inflammation type	1	Primarily lymphocytic
		2	Neutrophilic
		3	Lymphoplasmacytic

		4	Granulomatous
Gran_tiss	SM granulation tissue	1	No
		2	Yes
Lymph_agg	SM lymphoid aggregates	1	No
		2	Yes
Det_inflamm	Detrusor inflammation present	1	No
		2	Yes
Det_fib	Detrusor fibrosis	1	No
		2	Yes
Ser_inflamm	Serosal inflammation	1	No
		2	Yes
Ser_hem	Serosal hemorrhage	1	No
		2	Yes
Vasculitis	Presence of vasculitis	1	No
		2	Yes
Hyperemia	Presence of hyperemia	1	No
		2	Yes
Organisms	Presence of microorganisms	1	No
		2	Yes

LPF - low power field (100x magnification); MUSVM - School of Veterinary Medicine College of Science, Health, Engineering and Education, Murdoch University; SM - submucosa; UQVLS - University of Queensland Veterinary Laboratory Service.

Supplementary Table 2: Breakdown of pathology records where the diagnosis for the bladder tissue was assigned to the 'other' category (n = 47).

Species	Category	Pathology report diagnosis	n
Canine n = 34	No definitive diagnosis	Hemorrhage and/or edema	9
		Congestion	4
		Not reported	1
	Systemic disease	Peritonitis	3
		Coagulopathy	2
		Vasculitis	1
		Neospora caninum smooth muscle myositis	1
	Trauma	Trauma and bladder rupture	5
	Preservation artefact	Autolysis only	4
	Iatrogenic	Healing surgical wound	1
		Trauma from catheterization	1
	Other	Bladder polyp	1
		Papillary epithelial hyperplasia	1
Feline n = 13	No definitive diagnosis*	Focal hemorrhage	3
		Hemorrhage and edema	2
		Congestion only	2
		Smooth muscle hypertrophy	1
	Systemic disease	Focal hemorrhage, suspected coagulopathy	2
		Feline infectious peritonitis	1
	Trauma	Trauma and bladder rupture	1
	Anatomic	Cyst	1

*Potential feline idiopathic cystitis cases

Supplementary Table 3: Neoplasia types.

Species	Type of neoplasia	Count
Dogs n = 74	Urothelial cell carcinoma	53
	Leiomyoma/leiomyosarcoma	6
	Poorly differentiated carcinoma	4
	Lymphoma	2
	Fibropapilloma/fibroma	3
	Hemangiosarcoma	2
	Not definitive	4
Cats n = 10	Urothelial cell carcinoma	7
	Lymphoma	3

Supplementary Table 4: Canine predicted probabilities for each diagnosis by the five significant histological variables.

**Variables**					**Normal/Other**		**Cystitis**		**Neoplasia**		**Urolithiasis**	
uroth_ ulcer	lymph_ aggregates	sm_inflam_ type_2	uroth_ inflam	sm_hem	Prob	CI	Prob	CI	Prob	CI	Prob	CI
no	no	no	no	mild	0.71	0.53 - 0.90	0.08	0 - 0.15	0.21	0.05 - 0.36	not_sig	not_sig
no	no	no	no	moderate	0.65	0.42 - 0.88	0.14	0.01 - 0.28	0.21	0.03 - 0.39	not_sig	not_sig
no	no	no	no	severe	0.62	0.33 - 0.91	0.3	0.04 - 0.57	not_sig	not_sig	not_sig	not_sig
no	no	no	yes	mild	0.32	0.02 - 0.62	not_sig	not_sig	0.5	0.21 - 0.8	not_sig	not_sig
no	no	no	yes	moderate	not_sig	not_sig	0.26	0.02 - 0.5	0.46	0.16 - 0.76	not_sig	not_sig
no	no	no	yes	severe	not_sig	not_sig	0.57	0.22 - 0.91	not_sig	not_sig	not_sig	not_sig
no	no	yes	no	mild	0.38	0.15 - 0.62	0.27	0.08 - 0.47	0.31	0.09 - 0.53	not_sig	not_sig
no	no	yes	no	moderate	0.29	0.07 - 0.52	0.41	0.16 - 0.67	0.27	0.05 - 0.49	not_sig	not_sig
no	no	yes	no	severe	0.22	0 - 0.43	0.69	0.44 - 0.93	not_sig	not_sig	not_sig	not_sig
no	no	yes	yes	mild	not_sig	not_sig	0.34	0.08 - 0.61	0.47	0.17 - 0.76	not_sig	not_sig
no	no	yes	yes	moderate	not_sig	not_sig	0.49	0.18 - 0.79	0.38	0.08 - 0.67	not_sig	not_sig
no	no	yes	yes	severe	not_sig	not_sig	0.78	0.56 - 1.00	not_sig	not_sig	not_sig	not_sig
no	yes	no	no	mild	0.33	0 - 0.66	0.23	0 - 0.45	0.44	0.13 - 0.75	not_sig	not_sig
no	yes	no	no	moderate	not_sig	not_sig	0.35	0.05 - 0.65	0.39	0.08 - 0.69	not_sig	not_sig
no	yes	no	no	severe	not_sig	not_sig	0.66	0.31 - 1.02	not_sig	not_sig	not_sig	not_sig
no	yes	no	yes	mild	not_sig	not_sig	0.27	0 - 0.54	0.63	0.33 - 0.93	not_sig	not_sig
no	yes	no	yes	moderate	not_sig	not_sig	0.4	0.06 - 0.75	0.53	0.18 - 0.88	not_sig	not_sig
no	yes	no	yes	severe	not_sig	not_sig	0.78	0.48 - 1.07	not_sig	not_sig	not_sig	not_sig
no	yes	yes	no	mild	not_sig	not_sig	0.47	0.18 - 0.77	0.4	0.12 - 0.69	not_sig	not_sig
no	yes	yes	no	moderate	not_sig	not_sig	0.62	0.32 - 0.92	0.3	0.02 - 0.58	not_sig	not_sig
no	yes	yes	no	severe	not_sig	not_sig	0.88	0.72 - 1.00	not_sig	not_sig	not_sig	not_sig
no	yes	yes	yes	mild	not_sig	not_sig	0.47	0.14 - 0.79	0.48	0.15 - 0.81	not_sig	not_sig
no	yes	yes	yes	moderate	not_sig	not_sig	0.61	0.28 - 0.94	0.35	0.03 - 0.67	not_sig	not_sig
no	yes	yes	yes	severe	not_sig	not_sig	0.89	0.73 - 1.00	not_sig	not_sig	not_sig	not_sig

yes	no	no	no	mild	0.69	0.45 - 0.94	not_sig	not_sig	0.18	0 - 0.36	not_sig	not_sig
yes	no	no	no	moderate	0.63	0.37 - 0.89	0.15	0 - 0.3	0.18	0 - 0.36	not_sig	not_sig
yes	no	no	no	severe	0.57	0.31 - 0.83	0.31	0.09 - 0.54	not_sig	not_sig	not_sig	not_sig
yes	no	no	yes	mild	not_sig	not_sig	not_sig	not_sig	0.39	0.06 - 0.72	not_sig	not_sig
yes	no	no	yes	moderate	not_sig	not_sig	0.26	0.01 - 0.5	0.36	0.06 - 0.67	not_sig	not_sig
yes	no	no	yes	severe	not_sig	not_sig	0.49	0.17 - 0.8	not_sig	not_sig	not_sig	not_sig
yes	no	yes	no	mild	0.28	0.02 - 0.53	0.22	0.03 - 0.42	not_sig	not_sig	not_sig	not_sig
yes	no	yes	no	moderate	0.22	0.01 - 0.44	0.35	0.1 - 0.6	not_sig	not_sig	not_sig	not_sig
yes	no	yes	no	severe	0.15	0 - 0.3	0.53	0.27 - 0.8	not_sig	not_sig	0.27	0 - 0.54
yes	no	yes	yes	mild	not_sig	not_sig	not_sig	not_sig	not_sig	not_sig	0.56	0.07 - 1.00
yes	no	yes	yes	moderate	not_sig	not_sig	not_sig	not_sig	not_sig	not_sig	0.46	0 - 0.92
yes	no	yes	yes	severe	not_sig	not_sig	0.43	0.05 - 0.82	not_sig	not_sig	0.5	0.07 - 0.92
yes	yes	no	no	mild	not_sig	not_sig	0.25	0.01 - 0.49	0.39	0.07 - 0.71	not_sig	not_sig
yes	yes	no	no	moderate	not_sig	not_sig	0.38	0.08 - 0.68	0.34	0.05 - 0.63	not_sig	not_sig
yes	yes	no	no	severe	not_sig	not_sig	0.67	0.37 - 0.98	not_sig	not_sig	not_sig	not_sig
yes	yes	no	yes	mild	not_sig	not_sig	0.29	0 - 0.57	0.54	0.2 - 0.88	not_sig	not_sig
yes	yes	no	yes	moderate	not_sig	not_sig	0.42	0.09 - 0.76	0.45	0.1 - 0.79	not_sig	not_sig
yes	yes	no	yes	severe	not_sig	not_sig	0.74	0.46 - 1.00	not_sig	not_sig	not_sig	not_sig
yes	yes	yes	no	mild	not_sig	not_sig	0.44	0.15 - 0.73	0.3	0.03 - 0.58	not_sig	not_sig
yes	yes	yes	no	moderate	not_sig	not_sig	0.6	0.31 - 0.88	not_sig	not_sig	not_sig	not_sig
yes	yes	yes	no	severe	not_sig	not_sig	0.8	0.59 - 1.00	not_sig	not_sig	not_sig	not_sig
yes	yes	yes	yes	mild	not_sig	not_sig	0.37	0.04 - 0.7	not_sig	not_sig	not_sig	not_sig
yes	yes	yes	yes	moderate	not_sig	not_sig	0.52	0.18 - 0.86	not_sig	not_sig	not_sig	not_sig
yes	yes	yes	yes	severe	not_sig	not_sig	0.71	0.37 - 1.00	not_sig	not_sig	not_sig	not_sig

Key: CI - confidence interval; lymph_aggregates - lymphoid aggregates; not_sig - not significant; Prob - probability; sm_hem - submucosal hemorrhage; sm_inflamm_type_2 - type 2 (neutrophilic) submucosal inflammation; uroth_inflam - urothelial inflammation; uroth_ulcer - urothelial ulceration.

Supplementary Table 5: Feline predicted probabilities for each diagnosis by the five significant histological variables.

Variables					Normal/Other		Cystitis		Neoplasia		Urolithiasis	
uroth_ ulcer	lymph_ aggregates	sm_inflam_ type_2	uroth_ inflam	sm_hem	Prob	CI	Prob	CI	Prob	CI	Prob	CI
no	no	no	no	mild	0.55	0.42 - 0.68	0.12	0.05 - 0.19	0.32	0.2 - 0.44	not_sig	not_sig
no	no	no	no	moderate	0.48	0.3 - 0.65	0.2	0.09 - 0.32	0.31	0.16 - 0.46	not_sig	not_sig
no	no	no	no	severe	0.45	0.19 - 0.7	0.43	0.19 - 0.67	not_sig	not_sig	not_sig	not_sig
no	no	no	yes	mild	0.18	0.01 - 0.35	0.18	0.06 - 0.3	0.59	0.4 - 0.78	not_sig	not_sig
no	no	no	yes	moderate	not_sig	not_sig	0.29	0.11 - 0.46	0.53	0.32 - 0.73	not_sig	not_sig
no	no	no	yes	severe	not_sig	not_sig	0.62	0.36 - 0.88	not_sig	not_sig	not_sig	not_sig
no	no	yes	no	mild	0.22	0.09 - 0.35	0.31	0.18 - 0.44	0.37	0.23 - 0.51	0.1	0.01 - 0.18
no	no	yes	no	moderate	0.17	0.04 - 0.29	0.46	0.29 - 0.63	0.31	0.16 - 0.46	not_sig	not_sig
no	no	yes	no	severe	not_sig	not_sig	0.72	0.54 - 0.9	not_sig	not_sig	not_sig	not_sig
no	no	yes	yes	mild	not_sig	not_sig	0.31	0.14 - 0.47	0.44	0.25 - 0.63	0.21	0.04 - 0.38
no	no	yes	yes	moderate	not_sig	not_sig	0.45	0.25 - 0.64	0.36	0.17 - 0.55	0.16	0.01 - 0.3
no	no	yes	yes	severe	not_sig	not_sig	0.7	0.49 - 0.92	not_sig	not_sig	0.18	0 - 0.37
no	yes	no	no	mild	not_sig	not_sig	0.26	0.09 - 0.44	0.53	0.3 - 0.76	not_sig	not_sig
no	yes	no	no	moderate	not_sig	not_sig	0.39	0.16 - 0.62	0.45	0.21 - 0.69	not_sig	not_sig
no	yes	no	no	severe	not_sig	not_sig	0.73	0.47 - 1	not_sig	not_sig	not_sig	not_sig
no	yes	no	yes	mild	not_sig	not_sig	0.27	0.08 - 0.47	0.66	0.45 - 0.87	not_sig	not_sig
no	yes	no	yes	moderate	not_sig	not_sig	0.4	0.16 - 0.64	0.55	0.3 - 0.8	not_sig	not_sig
no	yes	no	yes	severe	not_sig	not_sig	0.78	0.53 - 1.03	not_sig	not_sig	not_sig	not_sig
no	yes	yes	no	mild	not_sig	not_sig	0.48	0.28 - 0.67	0.43	0.23 - 0.62	not_sig	not_sig
no	yes	yes	no	moderate	not_sig	not_sig	0.62	0.42 - 0.83	0.31	0.12 - 0.51	not_sig	not_sig
no	yes	yes	no	severe	not_sig	not_sig	0.88	0.74 - 1.01	not_sig	not_sig	not_sig	not_sig
no	yes	yes	yes	mild	not_sig	not_sig	0.44	0.23 - 0.64	0.47	0.25 - 0.68	not_sig	not_sig
no	yes	yes	yes	moderate	not_sig	not_sig	0.58	0.37 - 0.8	0.35	0.14 - 0.56	not_sig	not_sig
no	yes	yes	yes	severe	not_sig	not_sig	0.85	0.69 - 1.02	not_sig	not_sig	not_sig	not_sig
yes	no	no	no	mild	0.48	0.24 - 0.71	0.11	0.02 - 0.21	0.25	0.08 - 0.42	0.16	0 - 0.32

yes	no	no	no	moderate	0.42	0.2 - 0.64	0.2	0.06 - 0.33	0.24	0.08 - 0.41	0.14	0 - 0.28
yes	no	no	no	severe	0.35	0.14 - 0.56	0.37	0.18 - 0.57	not_sig	not_sig	0.2	0.03 - 0.38
yes	no	no	yes	mild	not_sig	not_sig	0.13	0 - 0.26	0.34	0.08 - 0.61	0.41	0.07 - 0.75
yes	no	no	yes	moderate	not_sig	not_sig	0.22	0.04 - 0.4	0.33	0.09 - 0.56	0.35	0.06 - 0.64
yes	no	no	yes	severe	not_sig	not_sig	0.38	0.12 - 0.64	not_sig	not_sig	0.46	0.15 - 0.76
yes	no	yes	no	mild	not_sig	not_sig	0.16	0.02 - 0.29	0.15	0.01 - 0.29	0.59	0.32 - 0.86
yes	no	yes	no	moderate	not_sig	not_sig	0.27	0.08 - 0.45	0.14	0.02 - 0.27	0.5	0.23 - 0.77
yes	no	yes	no	severe	not_sig	not_sig	0.38	0.14 - 0.61	not_sig	not_sig	0.54	0.27 - 0.80
yes	no	yes	yes	mild	not_sig	not_sig	0.09	-0.03 - 0.22	not_sig	not_sig	0.78	0.54 - 1.00
yes	no	yes	yes	moderate	not_sig	not_sig	0.17	-0.01 - 0.34	not_sig	not_sig	0.71	0.44 - 0.98
yes	no	yes	yes	severe	not_sig	not_sig	0.23	0.01 - 0.46	not_sig	not_sig	0.74	0.49 - 0.98
yes	yes	no	no	mild	not_sig	not_sig	0.27	0.08 - 0.46	0.44	0.2 - 0.69	not_sig	not_sig
yes	yes	no	no	moderate	not_sig	not_sig	0.41	0.18 - 0.63	0.38	0.15 - 0.6	not_sig	not_sig
yes	yes	no	no	severe	not_sig	not_sig	0.69	0.45 - 0.93	not_sig	not_sig	not_sig	not_sig
yes	yes	no	yes	mild	not_sig	not_sig	0.25	0.05 - 0.45	0.49	0.22 - 0.76	not_sig	not_sig
yes	yes	no	yes	moderate	not_sig	not_sig	0.38	0.14 - 0.62	0.42	0.17 - 0.67	not_sig	not_sig
yes	yes	no	yes	severe	not_sig	not_sig	0.65	0.36 - 0.94	not_sig	not_sig	not_sig	not_sig
yes	yes	yes	no	mild	not_sig	not_sig	0.35	0.14 - 0.56	0.25	0.07 - 0.43	0.36	0.12 - 0.61
yes	yes	yes	no	moderate	not_sig	not_sig	0.5	0.28 - 0.73	0.2	0.05 - 0.36	0.26	0.04 - 0.49
yes	yes	yes	no	severe	not_sig	not_sig	0.68	0.39 - 0.96	not_sig	not_sig	not_sig	not_sig
yes	yes	yes	yes	mild	not_sig	not_sig	0.24	0.04 - 0.44	0.2	0.02 - 0.39	0.55	0.25 - 0.85
yes	yes	yes	yes	moderate	not_sig	not_sig	0.37	0.14 - 0.61	0.18	0.03 - 0.34	0.44	0.16 - 0.72
yes	yes	yes	yes	severe	not_sig	not_sig	0.51	0.17 - 0.85	not_sig	not_sig	0.45	0.1 - 0.8

Key: CI - confidence interval; lymph_aggregates - lymphoid aggregates; not_sig - not significant; Prob - probability; sm_hem - submucosal hemorrhage; sm_inflamm_type_2 - type 2 (neutrophilic) submucosal inflammation; uroth_inflam - urothelial inflammation; uroth_ulcer - urothelial ulceration.
